# Statin use is associated with reduced motor recovery after spinal cord injury

**DOI:** 10.1038/s41394-020-00378-y

**Published:** 2021-02-03

**Authors:** Erin M. Triplet, Isobel A. Scarisbrick

**Affiliations:** 1 Mayo Clinic Graduate School of Biomedical Sciences, Mayo Clinic Alix School of Medicine and the Mayo Clinic Medical Scientist Training Program-Rochester, Rochester, MN USA; 2 Department of Physical Medicine and Rehabilitation, Rehabilitation Medicine Research Center, Rochester, MN USA; 3 Department of Physiology and Biomedical Engineering, Rochester, MN 55905 USA

**Keywords:** Translational research, Spinal cord injury

## Abstract

**Study design:**

We completed retrospective analysis of statin use in individuals with neurologically significant spinal cord injury in a historical cohort study.

**Objective:**

Our objective was to establish the prevalence of cholesterol-lowering agent use following spinal cord injury (SCI) and to determine the impact on recovery of motor function.

**Setting:**

Patients enrolled in the Rochester Epidemiology Project in Olmsted County, Minnesota, USA from 2005 to 2018 were included in analysis.

**Methods:**

Exclusion criteria: age <18, comorbid neurological disease, prior neurological deficit, nontraumatic injury, survival <1 year, or lack of motor deficit. Demographics and cholesterol-lowering agent use in 83 individuals meeting all criteria were recorded. A total of 68/83 individuals were then assessed for change in function over the first 2 months after injury using the ISNCSCI motor subscore. Statistical comparison between control and statin groups was done by two-sided Chi-squared test or two-tailed Student’s *t* test. Generalized regression was performed to assess associations between independent variables and functional outcome.

**Results:**

30% of individuals with SCI had a prescription for a cholesterol-lowering agent. No significant differences were observed in severity of injury or demographic composition between groups. The change in motor subscore was reduced in the statin group compared to controls (*p* = 0.03, Mann–Whitney). Both severity of injury and statin were significant predictors of reduced motor recovery (*p* = 0.001, and *p* = 0.04, respectively).

**Conclusions:**

Both severity of SCI and statins were significant predictors of reduced motor recovery. Additional investigation is needed to address potential impact of statin-therapy in the context of CNS injury and repair.

## Introduction

The central nervous system (CNS) is one of the most cholesterol enriched organs [1], and cholesterol synthesis is required for growth of new axons and synapses [2, 3]. While under physiologic conditions the blood–brain barrier renders the CNS impermeable to cholesterol [4], following insult (such as spinal cord injury (SCI)) the loss of barrier integrity renders the brain sensitive to peripheral cholesterol levels [5], and opens a temporary window for transport of both peripheral cholesterol and pharmaceutical agents. Despite the crucial role of cholesterol in establishment of neurites and synapses, there is little clinical data available on the prevalence and effects of widely prescribed cholesterol-lowering agents in the setting of neurotrauma.

Multiple preclinical studies, including work from our lab, indicate that in addition to its role in development, cholesterol levels also influence repair in the CNS, with inhibition of cholesterol synthesis demonstrating a particularly detrimental impact on axon growth and neurite regeneration in vitro. Chronic delivery of Atorvastatin impaired cognitive function in mice, resulting in a significant decrease in post-synaptic densities [6]. In human studies, individuals with chronic SCI demonstrated a correlation between low levels of serum HDL and reduced motor function [7]. However, assessment years after injury made it impossible to determine whether dyslipidemia in individuals with SCI was a cause or consequence of reduced motor function. To address these questions, we initiated a retrospective chart-review study of adults with traumatic SCI to assess (1) frequency of statin use in the acute/subacute period post injury, and (2) whether statin use was associated with different rates of recovery.

## Methods

### Study design and population

We analyzed data available in the Olmsted County Rochester Epidemiology Project (REP) Database from 2005 to 2018 in a retrospective analysis of individuals presenting with SCI. The REP is a well-documented resource for retrospective studies; for a complete description of the methodology and infrastructure, refer to St Sauver, et al. [8]. A total of 1116 records were identified as having a relevant diagnostic code within the study period. For the present study, we excluded individuals <18 years of age at time of injury as well as those with comorbid neurological disease, previously existing focal neurological deficits, nontraumatic mechanism of injury, survival <1-year post injury, and individuals without documented neurological deficits, resulting in 83 participants for subsequent analysis. Detailed ethnographic breakdown is not provided for the control and statin-treated subpopulations due to the small study size and to protect patient privacy. The study was approved by the Institutional Review Boards at both Mayo Clinic and Olmsted Medical Center; only patients who had provided written consent for their data to be used for research purposes were accessed in the course of this study.

### Data collection

Medical records from the study population were analyzed for demographic information (sex, race/ethnicity, age at time of injury) as well as prescribed cholesterol-lowering agents. Cholesterol-lowering agents assessed include HMG CoA Reductase inhibitors (statins), cholesterol uptake inhibitors (ezetimibe, cholestyramine), and fibrates. Records were also assessed to determine timing of cholesterol-lowering agent initiation relative to SCI. In all 25 individuals (30% of population) with noted cholesterol-lowering agents documented, onset of therapy preceded SCI and continued to be documented in the record during the acute recovery phase. As the majority (24/25, *n* = 1 cholesterol uptake inhibitor) of individuals were treated with a statin, this subpopulation is referred to as the “Statin” group.

A total of 68/83 of the study population were then further assessed for functional recovery following SCI where sufficiently detailed records at both baseline (time of injury) and at 2 months follow-up (7–10 weeks post injury) permitted. Functional impact was assessed using the International Standards for Neurological Classification of Spinal Cord Injury (ISNCSCI) motor subscore as a quantitative indicator of motor function. When ISNCSCI motor score was not recorded directly, given motor function scores were converted to the ISNCSCI system by a trained data abstractor. This scale is a widely accepted scoring system for assessing SCI; briefly, scores of 0–5 (0 = total paralysis, 5 = full function) are assigned to five major motor functions in each limb, for a total possible score of 100 (no motor deficit). Individuals were scored both at presentation and at 2-month follow-up, and this difference recast as change in motor score (motor subscore at 2 months − motor subscore at injury) as the primary metric of acute functional recovery. “Percent recovery” was defined as:

$$\frac{\left( {\mathrm{Score}}\,{\mathrm{at}}\,2\,{\mathrm{months}} - {\mathrm{Initial}}\,{\mathrm{Score}} \right)}{{\mathrm{Max}}\,{\mathrm{Possible}}\,{\mathrm{Score}}} - ({\mathrm{Initial}}\,{\mathrm{Score}}\times\,100)$$

### Statistical analysis

All statistical analysis was performed using JMP Pro (SAS, Cary, NC). Statistical comparison between control and statin-treated groups was done by two-sided Chi-squared test (for categorical variables) and two-tailed Student’s *t* test or Mann–Whitney nonparametric test (for continuous variables, Mann–Whitney for smaller datasets); specific tests used are indicated in individual figure legends. Generalized regression with lasso adaptation modeling was performed to assess associations between independent variables (age at injury, sex, severity of injury at presentation, and cholesterol-lowering agent use status) and functional outcome (change in ISNCSCI motor subscore). Further full factorial analysis was not performed due to the small sample size.

## Results

We analyzed the medical records of patients with SCI to establish prevalence of statin use and possible impact on functional outcomes. This analysis produced a dataset of 83 individuals who presented with SCI, of whom 31% (26/83) were female, with an average age at time of injury of 49 (range: 18–88) (Table 1). The study population was 89% white, 4.8% black/African American, 2.4% Hispanic/Latino, and <1% American Indian/Alaska native, Asian, and other. These metrics are reasonably well aligned with national demographics of SCI patients [9], and taken to suggest that our study population can reasonably be used as a representative group.

**Table 1:** Statin therapy is common in SCI.

	Ctrl	Statin	*p* value
Percentage	70% (58/83)	30% (25/83)	–
Male/Female	37/21 (36% ♀)	20/5 (20% ♀)	0.09
% White	90% (52/58)	88% (22/25)	0.79
Age in years	41.0 (±2.5)	66.8 (±3.3)	<0.001*
Motor score at injury	63.5 (±4.1)	71.6 (±7.7)	0.33
Motor score at follow-up	78.4 (±3.5)	77.1 (±6.9)	0.86
Change in motor score	14.9 (±2.3)	5.5 (±2.8)	0.02*

Analysis of medical records of SCI patients demonstrates that 30% of individuals presenting with SCI and neurological deficit have a current prescription for a cholesterol-lowering agent (*n* = 24 statin, *n* = 1 cholesterol uptake inhibitor). In all cases, the statin prescription was initiated before injury. Functional status was assessed by scoring motor function using the ISNCSCI motor subscore (0–100, 100 = full motor function in periphery, 0 = total paralysis) based on status at the time of injury and at 2-month follow-up (range 7–10 weeks). The statin-treatment group was significantly older at the time of injury (mean 66 vs. 45, *p* < 0.01). The control group showed a trend toward poorer functional status at time of injury; neither score at injury or follow-up differed significantly between groups. The change in motor subscore (score at follow-up − score at injury), a measure of functional improvement, was significantly worse in the statin-prescribed group compared to the controls (*p* = 0.02). Significant differences between statin and control groups determined by two-way Chi-squared test (sex, race/ethnicity) or two-tailed Student’s *t* test (age, motor scores), *p < 0.05. Data for age and motor scores are shown as mean ± S.E.M.

Bold values indicate statistical significance *p* < 0.05.

Among the 83 individuals included in the study population, 30% had a current prescription for a cholesterol-lowering agent at time of injury (*n* = 24 statin class, *n* = 1 cholesterol uptake inhibitor—grouped as the “Statin” cohort). Comparison between the control and statin-treated groups revealed a significant difference in age at time of injury (ctrl = 41.0, statin = 66.8, *p* < 0.01, Student’s *t* test). Severity of injury was comparable between the groups (*p* = 0.33). We also found a significant difference between the change in motor subscore (*p* = 0.03), with the control group demonstrating an average improvement of 15 points compared to 5.5 in the statin-treated group (Table 1).

**Fig. 1:**
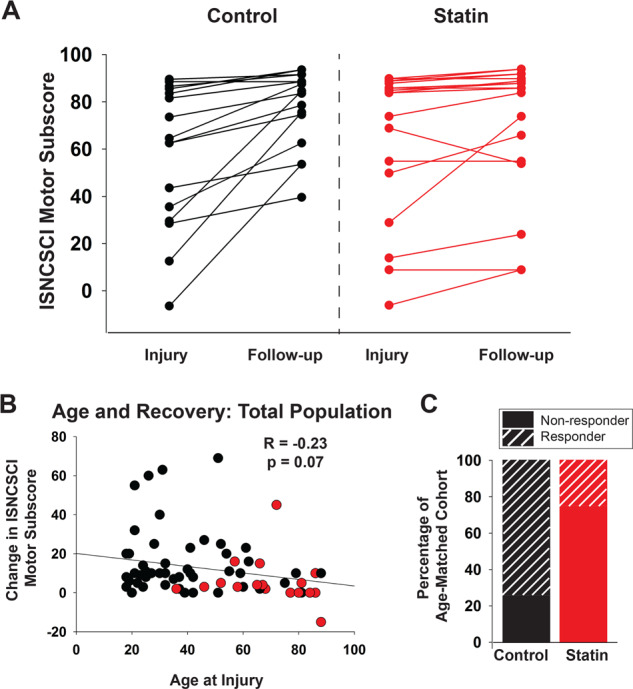
Statin use is associated with diminished recovery in an age-matched cohort. **A** Graph shows paired ISNCSCI motor subscores at time of injury and at 2 months recovery for all individuals in the age-matched cohort (see Table 2), and demonstrates loss of substantial improvement in statin group. **B** There was a mild association between age and improvement on motor subscores, Pearson *R* = −0.23, *p* = 0.07 (not significant) in the complete study population, although age was not a significant predictor of functional improvement in generalized linear regression. Motor subscore at injury was the most significant factor in predicting change in motor score by generalized linear regression analysis (*p* < 0.01), statin also demonstrated a significant impact on change in motor score (*p* = 0.04). In the age-matched cohort, patients on cholesterol reduction therapy were significantly less likely to achieve increases in motor score of 10 or greater (“responders”) compared to the statin-treated group, *p* = 0.004, Chi-Square test, OR = 0.12, 95% CI 0.03–0.55 (**C**). Relative contributions of independent variables (sex, age at injury, motor subscore at injury, and statin usage status) to motor recovery (change in motor subscore) were quantified by general linear regression.

Age is a well-documented factor influencing recovery, with increasing age associated with reduced capacity for repair [10]. We observed a mild correlation between age at injury and motor improvement (Pearson *R* = −0.23, *p* = 0.07, Fig. 1B). To account for the impact of the relative youth of the control group, we repeated analysis comparing the statin cohort (*n* = 18) to a subset of the control cohort (*n* = 17) which included all individuals >40 years of age with two younger individuals age- and sex-matched to the statin-treated individuals <40. These groups had better parity in age—control: mean 54 (range: 26–88) vs. statin: mean 66 (range: 20–88) (*p* = 0.06, Mann–Whitney nonparametric test) as well as severity of injury—ISNCSCI motor subscore of 53.7 vs. 66.1 at time of injury (*p* = 0.26) (Table 2). In these comparatively age-matched cohorts, the control group still demonstrated significant increases in motor recovery, with over three-fold greater change in motor subscore (17.7 vs. 5.8 in the statin group, *p* = 0.003).

**Table 2:** Statin use is associated with diminished recovery.

	Ctrl	Statin	*p* value
Number	17	18	–
Male/Female	15/2 (11% ♀)	17/1 (5% ♀)	0.37
% White	100% (17/17)	83% (15/18)	–
Age in years	53.7 (±4.6)	66.1 (±4.4)	0.06
Motor score at injury	65.4 (±7.1)	71.6 (±7.7)	0.26
Motor score at follow-up	83.1 (±4.0)	77.1 (±6.9)	0.99
Change in motor score	17.7 (±4.4)	5.5 (±2.8)	0.003*

To further analyze the potential impact of cholesterol-lowering therapy while minimizing any confounding effects of age, an age-matched subset was randomly selected from the control group for subsequent analysis. The mean age of the statin group was 66.1 years (range: 20–88); for control, 53.7 (range: 26–88)—this difference was not statistically significant (*p* = 0.06). Even in comparison to the older control population, the statin-treated individuals showed significantly less functional motor recovery. The control group demonstrated greater than three-fold increased change in ISNCSCI motor subscore (*p* = 0.003) at 2-month follow-up. *p < 0.05. Significant differences between statin and control groups determined by two-way Chi-squared test (sex) or Mann–Whitney nonparametric test (age, motor scores). Comparison could not be made on racial/ethnic background due to lack of non-Caucasian individuals in the control group. Data for age and motor scores are shown as mean + S.E.M.

Bold values indicate statistical significance *p* < 0.05.

Generalized regression was used on this subset to assess contributions of the independent variables of age at injury, sex, severity of injury, and cholesterol-lowering agent use to the change in motor score. We found that severity of injury was the strongest predictor of recovery (*p* < 0.01). Cholesterol-lowering agent use was also a significant predictor of change in motor score (*p* = 0.04). To address the ceiling effect (i.e. patients with milder injury had limited possible improvement), we also calculated percent recovery, and performed the same analysis. Again, generalized regression showed that severity of injury was the strongest predictor of recovery (*p* = 0.03) with cholesterol-lowering agent use also bordering on significance (*p* = 0.052). Based on assessment of the paired distributions of scores (Fig. 1A) at injury and follow-up, it is apparent that statin-treated patients with very mild SCI tended to recover as well as the control group, however, in cases of moderate to severe injury, individuals prescribed a cholesterol-lowering agent were less likely to make dramatic improvements in the 2 months following their injury. This is a crucial observation, as studies have repeatedly demonstrated that once progress plateaus in the first year after SCI, there is almost no further functional improvement. [11], barring future advances in regenerative therapy. To quantify this observation, we classified study participants as “responders” or “non-responders,” with responders defined as patients that showed improvement in the ISNCSCI motor subscore ≥10 points. Despite similar distributions of injury severity, patients on cholesterol reduction therapy were significantly less likely to achieve increases in motor score of 10 or greater (*p* = 0.004, Chi-Square test, OR = 0.12, 95% CI 0.03–0.55).

## Discussion

Use of statins is exceedingly common in the Western world. Atorvastatin (Lipitor) is the single most prescribed agent in the United States based on data of prescriptions filled at pharmacies nationwide [12], and overall 1 in 4 American adults is currently prescribed a statin [13]. As concerns of dyslipidemia and cardiovascular risk continue to rise, it is evident that widespread statin use will persist, making understanding the possible neurological consequences of statin use even more pertinent.

In the present study, we identified that a third of all adults with traumatic SCI present while currently prescribed a cholesterol-lowering agent, a percentage which progressively increases with age. Continuation of statins on hospital orders for SCI patients who received inpatient treatment indicate that statin use is not routinely discontinued after injury. Statin use was associated with reduced motor recovery, and individuals prescribed a statin were less likely to show significant improvement (change in motor score ≥10 points). Both score at injury and statin use were significant predictors of recovery; lower motor score at injury was associated with greater recovery (with a maximum score of 100 on the ISNCSCI scale, patients who presented with a relatively minor injury had a limit on the degree of change possible), and statin use was a predictor of limited improvement.

Concerns about the potential negative neurological side-effects of statin therapy are relatively new; in 2012, the FDA issued a black-box warning for cognitive decline, depression, and memory loss/confusion, an issue still under debate [14, 15]. This action was taken in response to widespread clinical reports of cognitive dysfunction temporally associated with statin treatment, in spite of the failure of large-scale meta-analysis to detect a significant impact of statin therapy on cognitive outcomes [16]. It is also necessary to consider the pleiotropic nature of statins’ physiological activities in vivo, including inhibition of HMG-CoA Reductase, Stat3 inhibition and anti-inflammatory properties, among others [17]. The multitude of potential physiological actions makes it difficult, if not impossible, to directly link the impact of statins on neurological function directly to its role in cholesterol synthesis.

There are several limitations to the interpretation of findings in the current study. First, although we demonstrate association between statin therapy and reduced functional recovery following SCI, there are multiple unassessed covariables which may explain some or all of this relationship. Statin therapy may be an indicator of poor health overall, limiting the impact of physical therapy and/or endogenous repair capacity. Recovery of function also continues beyond the subacute period defined in this study [11]; however, inconsistent follow-up at later timepoints precluded assessment of long-term progress in this data set. As such, it is not clear whether any potential impact of statins may be limited to the early recovery period. Understanding the timeline of statins’ impact on neurological repair is crucial, as cardiovascular disease is a leading cause of death in the SCI population [18], and statin use is associated with reduced mortality over the long term [19]. Statins have well-documented pleiotropic effects beyond their impact on cholesterol synthesis, including anti-inflammatory properties which have provided modest benefit in some rodent studies [20, 21]. Therefore, it is not possible from this dataset to conclude the degree to which inhibition of cholesterol biosynthesis impacts neural repair. Future studies are needed to determine the impact peripheral cholesterol levels may play in the context of injury and repair in the CNS. While all the individuals assessed for functional recovery had some physical therapy in the period following their injury, it is not possible to assess the degree of participation, which may further impact the degree of recovery, in addition to a multitude of other factors, such as socioeconomic status and social support.

In this retrospective chart-review study of individuals with neurologically significant SCI in Olmsted County, severity of injury and concurrent cholesterol-lowering agent therapy were predictors of functional recovery. Further larger-scale investigation is needed to assess the role of serum lipids (HDL, LDL, etc.) in modulating CNS recovery after injury and to provide further information on the impact of statins and other cholesterol-lowering agents in the acute period following traumatic injury to the CNS.
